# Development and preliminary validation of the Danish headache questionnaire

**DOI:** 10.1186/s12998-025-00573-4

**Published:** 2025-02-27

**Authors:** K. B. Dissing, R. K. Jensen, H. W. Christensen, M. E. Jensen, H. H. Lauridsen

**Affiliations:** 1 Chiropractic Knowledge Hub, Odense, Denmark; 2 Department of Sports Science and Clinical Biomechanics, University of Southern Denmark, Odense, Denmark; 3 Private chiropractic practice, Copenhagen, Denmark

**Keywords:** Headache, Questionnaire, Management, Primary care, Prevalence, Chiropractic

## Abstract

**Background:**

The prevalence of headache disorders is imposing a growing burden on public health. Although most patients are seen in primary care, there is an absence of validated questionnaires designed to describe how clinicians manage patients with headache in primary care. The aim of this study was to develop a standardised headache questionnaire for use by primary care clinicians, covering diagnostic procedures, management strategies, and treatment modalities, and to assess the prevalence of consultations for headache in primary care.

**Methods:**

The Danish Headache Questionnaire was developed through a three-phase process: a development phase, a content validation phase via iterative feedback, and a phase to create a generic English version. The Danish Headache Questionnaire includes a survey that covers diagnostic procedures, management strategies, and treatment modalities, and a logbook for tracking the prevalence of consultations for headaches. The questionnaire was tested by Danish chiropractors in primary care from 2020 to 2022.

**Results:**

The Danish Headache Questionnaire underwent several modifications. The survey was expanded to include questions about the Danish profession-specific guideline for managing headaches, different headache types, medical history, radiographic imaging, and potential side effects. The logbook was revised to allow for the documentation of multiple headaches and included a separate form for recording the total number of consultations. The generic version was adapted by removing or adjusting profession-specific terms and questions to suit other clinical environments. The final Danish Headache Questionnaire is available in a generic and a chiropractic-specific format, and was translated to English through a cross-cultural adaptation process.

**Conclusions:**

The Danish Headache Questionnaire has good content validity and is a feasible tool for assessing clinicians’ knowledge in managing patients with headaches and gathering data on headache prevalence in primary care. The generic version promotes a uniform approach and enables comparative analysis across different settings. The Danish Headache Questionnaire may be a valuable instrument guiding teaching a standardised assessment and for clinical assessment in primary care. Furthermore, it may have the potential to fill in gaps of knowledge which could improve the management of headache disorders in primary care.

**Supplementary Information:**

The online version contains supplementary material available at 10.1186/s12998-025-00573-4.

## Background

In recent years, there has been a growing awareness of patients experiencing headaches, and headache disorders have imposed a growing burden on public health, impacting both personal well-being and socioeconomic aspects [1, 2]. Headache rank as the third leading cause of global disability, and as the primary contributor for individuals under the age of 50^2^. In Denmark, a survey from 2021 including more than 170.000 citizens reported that 18% suffered from migraine or frequent headache, predominantly affecting women [3]. Primary care settings serve as the primary point of contact for patients with headaches, with the general practitioner being the primary health care provider managing the course of treatment. Notably, one in three people has sought help for headache in primary care at some point in their lives [4]. In Denmark, the knowledge about the prevalence of patients with headaches in general practice is sparse, but one study from general practice reported that 10% of their patients present with a migraine and 5% with a chronic headache [5]. In Denmark, chiropractors and physiotherapists are part of the primary health care system and offer non-pharmacological treatments for headache, such as patient education, physical activity and exercise, and manual therapy. To our knowledge, there is no data on the prevalence of patients with headaches in physiotherapy practice in Denmark. The knowledge of Danish chiropractors is also sparse, but a project by the authors of this study reports that approximately 12% of all patients in chiropractic practice present with headaches [6].

Current clinical guidelines and care standards [7–12] issued by governmental health agencies, non-governmental organisations, general practitioners, and chiropractors support non-pharmacological interventions and recommend managing patients with headaches in primary care whenever feasible. Moreover, these guidelines recommend that headache diagnoses should be based on the International Classification of Headache Disorders (ICHD-3) criteria for primary and secondary headaches [13], a classification of all headache-related disorders and their diagnostic criteria published by the International Headache Society.

Despite the availability of numerous headache guidelines, only few studies have investigated the extent to which healthcare professionals adhere to current guidelines in diagnosing and managing patients with headaches, and how these disorders are managed in primary care [10, 14, 15]. A global study conducted in 2011^1^ revealed that only half of the clinicians routinely incorporate guidelines and recommendations into their practices for diagnosing and treating headaches, emphasising the need for structured educational programs. Notably, most studies used self-developed, non-validated questionnaires, and to our knowledge, there exists no validated questionnaire specifically designed to assess the management of patients with headaches in primary care.

To address this shortage, this study aimed to develop a standardised headache questionnaire for primary care clinicians applicable across various settings, encompassing diagnostic procedures, management strategies, and treatment modalities. The specific objectives of this study were to (1) describe the development process of the Danish Headache Questionnaire, detailing its construction and refinement, (2) outline the validation process of the Danish Headache Questionnaire within a cohort of chiropractors, assessing its feasibility and content validity in capturing relevant data, and, (3) facilitate the translation and cross-culturally adaption of a finalised generic headache questionnaire into English, ensuring its linguistic and cultural appropriateness for a broader, English-speaking audience.

## Methods

The development of the Danish Headache Questionnaire (DHQ) comprised three distinct phases: an initial development phase, a subsequent content validation phase, and a final phase dedicated to creating a generic English version. All phases used COSMIN (Consensus-based Standards for the selection of health Measurement Instruments) as the main methodological framework [16].

### The development phase

The Danish Headache Questionnaire consists of two parts: a comprehensive survey targeted at the clinicians, and a logbook to register the prevalence of consultations for headaches. The project team of researchers and clinicians developed the first draft of the DHQ. The project team consisted of two chiropractors with less than one-year clinical experience and three experienced senior researchers with a chiropractic background and with four or more years of research experience. One of the team members is both a researcher and chiropractor in private practice with over 20 years of experience in managing patients with headaches. The development process comprised three key steps: (1) conducting a comprehensive literature search to identify existing questionnaires, (2) developing a survey for the Danish Headache Questionnaire, and (3) developing a logbook designed to systematically document the prevalence of consultations for headaches.

#### Literature search for existing questionnaires

In May 2020, a literature search, encompassing PubMed, relevant guidelines, and articles, was conducted to identify studies using headache questionnaires relevant to primary care. The research team conducted a focused search of PubMed and, in collaboration with a research librarian, of available guidelines in the field and used consensus to select the most relevant. The search terms included terminology relating to ‘chiropractic’, ‘headache’, ‘diagnosis’, and ‘questionnaire’. Among the retrieved studies, only one questionnaire aligned with the objectives of our study, namely, a questionnaire developed by Moore et al. [17] in a study involving Australian chiropractors. The authors of the Australian study developed a questionnaire that collected information on practitioner demographics, use of diagnostic criteria for headaches, headache management, and practitioner-reported prevalence of headaches in the preceding two weeks. The studied headaches included migraine, tension-type headaches and cervicogenic headaches. Subsequently, the lead author of the Australian study was contacted, and permission was obtained to adapt the questionnaire as the fundamental source of inspiration for the Danish Headache Questionnaire.

#### Development of the survey

The original Australian questionnaire consisted of six domains: practitioner characteristics, headache prevalence, headache classification, treatment outcome measures, multidisciplinary care, and chiropractic headache management, with a total of 31 questions. These questions were translated into Danish with minor adaptations. The initial translation was done by two master’s students in collaboration with two senior researchers from the project team. Additional questions were added to the translated version to cover topics not covered in the Australian version and to assess knowledge of the Danish profession-specific clinical care standard for the management of patients with headaches. The Danish Chiropractic Society published the care standard in 2019 to improve knowledge of headache and to standardise procedures and management within the Danish chiropractic community [11]. The standard includes recommendations for medical history, examination, diagnosis, and management.

The research group, which included a clinician, was responsible for developing all the supplementary questions.

Thus, the first version of the survey consisted of 12 domains with 45 questions on the following domains: (1) clinician characteristics, (2) prevalence of headache consultations, (3) knowledge of the Danish profession-specific clinical care standard, (4) knowledge of the categorisation and diagnosis of primary types of headaches, (5) knowledge of the classification and diagnosis of secondary types of headaches, (6) knowledge of the classification and diagnosis of other types of headaches, (7) medical history and physical examination items assessed by the clinician, (8) monitoring tools and treatment outcome measures used by the clinician, (9) collaboration with other health care providers, (10) treatment modalities and outcomes, 11) number of consultations, duration and frequency of care, and 12) perceived efficiency.

#### Development of the logbook

In the Australian questionnaire, the prevalence estimates were based on retrospective data from the past two weeks. We chose to have a four-week prospective registration period in order to obtain more representative estimates. In addition, we chose to register prospectively to minimise recall bias and to increase the accuracy of registrations. Instead of integrating the prevalence registration into the digital survey, a physical logbook for tracking the prevalence of consultations for headaches was developed as the registration tool. There was consensus in the project team of researchers and clinicians, that it would increase the likelihood of chiropractors remembering to register if there was a physical logbook visible on the office desk. During the four-week registration period, all patients with headaches were recorded by the chiropractors, as well as the total number of patients, regardless the diagnosis.

The logbook included the patient´s first name, date of birth, and type of consultation. The latter was categorised as new or existing patient. A new patient was defined as a patient presenting with headache who had never been to the clinic before or a patient already known in the clinic, but with headache as a new problem. An existing patient was defined as a patient who had already started a course of treatment for a headache diagnosis before the registration period.

It was also reported whether the headache was the primary or secondary reason for consulting a chiropractor. A secondary reason could be, for example, a headache in addition to neck- and shoulder complaints, or that the patient did not find the headache relevant to the consultation. At the end of the registration period, the total number of consultations for each patient was counted and recorded. All definitions were explained to the clinician in detail on the first page of the logbook.

### The content validation phase

#### Content validity of the Danish headache questionnaire

**Fig. 1:**
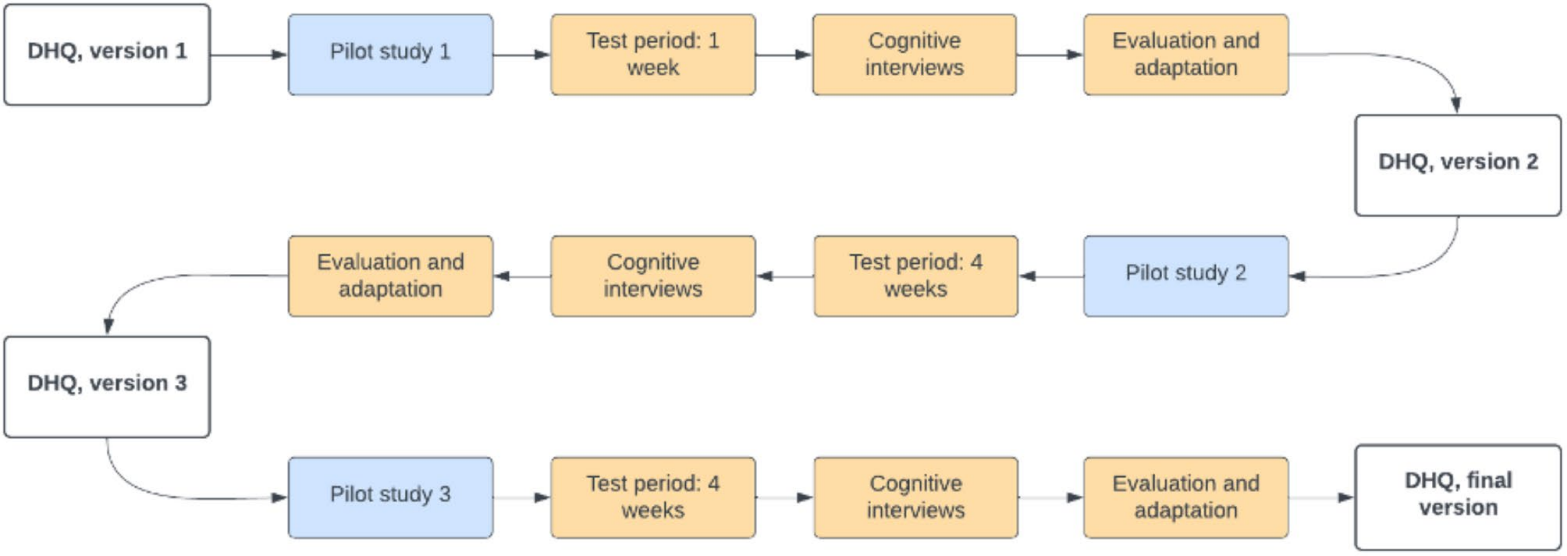
Three pilots shaping the development of the Danish Headache Questionnaire (DHQ) to its final form

The Danish Headache Questionnaire, comprising the survey and the logbook, was tested for content validity (i.e., item relevance, item comprehensiveness, and item comprehensibility) [16] using an iterative process. This involved three pilot tests in which the initial version of the DHQ was progressively drafted, evaluated, and revised to arrive at the final version (Fig. 1). A simplified version of the content validity method proposed by Patrick et al. was used to assess respondent understanding [18].

##### Data collection

The survey was administered digitally to the participating chiropractors. In pilot study 1 and 2, it was conducted using the SurveyXact platform [19], while pilot study 3 utilized the REDCap electronic data capture tool [20, 21] hosted by OPEN, Open Patient data Explorative Network, Odense University Hospital, Region of Southern Denmark [22]. This system ensures survey anonymity and confidentiality. It was possible to reply on a mobile phone, computer, or tablet. The estimated time to answer the questionnaire was 20–30 min. The response categories were binary (yes/no), multiple choice, categoric, and free text. There was an introductory page introducing the questionnaire and describing the content, estimated time consumption, and information about anonymity. Pilot study 1 and 2 were undertaken from September to November 2020, and pilot study 3 from January to February 2022.

##### Pilot study 1

In pilot study 1, five chiropractors from one local clinic from the clinical research network at the Chiropractic Knowledge Hub, Denmark, were invited to participate and four chiropractors accepted. Invitations were sent by email and included a consent form for participants to provide written informed consent. Initially, the participants registered patients for one week in the logbook. After completion, the chiropractors received an email with a link to the survey. Subsequently, semi-structured interviews on content and feasibility were performed with each participant, based on a predefined interview guide. The focus areas of the interview guide were on questionnaire instructions and format, recall, content understanding, response options, number of questions, and duration of answering. One interview was performed on site at the clinic, the other three were performed online using the Zoom platform due to responder preferences [23]. All interviews were recorded on an iPhone and transcribed verbatim. Based on the interviews and written feedback from the participants, the research group decided on revisions to both the survey and logbook resulting in version 2 of the DHQ.

##### Pilot study 2

A clinical research network at the Chiropractic Knowledge Hub, Denmark, was used for recruitment [24] in pilot study 2. The network consists of chiropractic clinics scattered throughout Denmark interested in participating in research activities. 107 chiropractors from the network were invited through email. A predefined number of 10–12 chiropractors was chosen based on the study by Moore et al. [14]. Participating chiropractors were admitted on the basis of the order of their response to the invitation. The registration of patients took place over four weeks whereafter the participating chiropractors received an email with a link to the survey. Subsequently, semi-structured interviews were carried out focusing on the participants experience with the logbook registration and understanding of certain aspects of the survey, that were pointed out in pilot study 1. Due to time constraints and resource limitations in this phase of the project, only two participants were interviewed. Both interviews were performed online using the Zoom platform. Despite the limited number of interviews, the data obtained provided significant insights into the aspects considered and the feedback from the two participants was consistent. Subsequent adaptations were performed resulting in the DHQ, version 3.

##### Pilot study 3

Invitations were sent by email to 23 chiropractors from four different clinics. The clinics were a convenience sample based on their prior interest in participating in research and not having participated in pilot study 1 and 2. Information meetings about the project were held either online using the Zoom platform or on site at the clinic for the convenience of the chiropractors before they agreed to participate. Nine chiropractors gave written informed consent to participate. Subsequently, semi-structured interviews on content and feasibility were conducted. Adequate feedback has been obtained with six participants as themes in the data became repetitive and additional interviews were not expected to provide more information. The interviews were based on a predefined interview guide focusing on understanding of content, number of questions, format, and duration of answering. Specifically, the interviews focused on format, duration, and content for the logbook. Pilot study 3 gave rise to minor adaptations and resulted in the final version of the DHQ.

### Development of a generic version of the Danish headache questionnaire

The DHQ was developed with the intension of being applicable in other countries and settings. Hence, a cross-cultural adaptation and translation of the final version of the survey and logbook into English was generated, following a modified version of the guideline developed by Beaton et al. [25]. Adapting and translating was done by two native English speakers and two native Danish speakers. Briefly, the final Danish version was forward translated by two bilingual translators (T1 and T2) with English as their native language, one a researcher with some knowledge of the field (also a chiropractor for over 15 years) and the other a layperson. The two translations were compared by the research team and a combined version was agreed upon (T12). The T12 version was backwards translated by two lay people (BT1 and BT2) with Danish as their native language, and good spoken and written English. An expert committee consisting of the research group, carried out a content validity check by comparing BT1, BT2 with the original text, changes were added to T12, and a final English version was agreed upon. Decisions were made by the research group, and any disagreements were discussed with the translators. The process also involved converting items into generic versions so the DHQ can be used by other health care professionals and researchers, who wants to explore management of patients with headaches. The generic version has been developed with comments and suggestions for alternative questions appropriate for other clinical settings.

## Results

### Development of the Danish headache questionnaire

Additional questions were incorporated into the translated edition to address issues not addressed in the Australian version and to encompass topics specific to Danish circumstances. In the practitioner characteristics section, items were added regarding the age of the chiropractor, and any additional educational qualifications. Furthermore, a question about the number of “existing patients” was added to the prevalence section, referring to patients already undergoing a course of treatment. We also included questions on use and knowledge of the Danish profession-specific clinical care standard for the management of patients with headaches in the Danish survey. An overview of all changes and additions in the Danish Headache Questionnaire (DHQ) compared to the Australian version is outlined in Table 1.

**Table 1:** Content of Danish headache questionnaire in first adapted Danish version versus Australian version

Domains	Australian version	Danish version	Domains added	Changes or additions
Practitioner characteristics	✓	✓		Age and other education added.
Headache prevalence	✓	✓		Number of “existing patients” added. A physical logbook was developed to register prevalence.
Knowledge of Danish profession-specific clinical care standard		✓		Added to enhance knowledge of headaches and to standardize procedures and management.
Headache classification	✓		• Primary types of headaches • Secondary types of headaches • Other types of headaches	This section was split into new sections: primary, secondary, and other types (see below).
Primary types of headaches		✓		More detailed questions added about the specific diagnoses. The diagnostic criteria added in detail.
Secondary types of headaches		✓		More detailed questions added about the specific diagnoses. The diagnostic criteria added in detail.
Other types of headaches		✓		Added as a separate section
Medical history and physical examination		✓		Added to gain more information about how patients with headaches are managed.
Treatment outcome measures	✓	✓		Two outcomes removed and one added.
Multidisciplinary care	✓	✓		Adapted to Danish setting.
Headache management	✓		• Treatment modalities and outcomes • Number of consultations, duration, and frequency of care • Perception of efficiency	This section was split into new sections (see below):
Treatment modalities and outcomes		✓		Minor changes to response options.
Number of consultations, duration, and frequency of care		✓		Minor changes to response options.
Perception of efficiency		✓		Added as a separate section

### Content validation of the Danish headache questionnaire

Based on the feedback from the participating chiropractors during the three pilot tests and discussions in the research group, some alterations to the DHQ were decided upon.

Initially, the survey was administered to the participants after they had completed the logbook for tracking the prevalence of consultations for headaches. Subsequently, the survey was modified so that completion of the survey was a prerequisite for logbook entry. This adjustment aimed to enhance participants’ comprehension of the diagnostic criteria for the most common primary and secondary headaches, which were included in the survey. Based on feedback from participating chiropractors, a separate form was added to register the total number of consultations, as well as the total number of consultations with new and existing patients.

As neck pain is often associated with headache [26, 27], it was important to know more about which spinal examinations, including imaging, are used to diagnose headache. Chiropractors often have access to x-rays in their own facilities. However, the frequency of spinal x-rays utilisation in patients with headaches remains unknown. Consequently, a question on this topic was added: “How often do you carry out an x-ray examination of the spine in patients with the following types of headaches (tension type, migraine, cervicogenic)?”.

We also added a question on experience of side effects: “Describe the most common side effects experienced by patients with headaches after treatment”, as this was not part of the Australian questionnaire and could provide relevant information to be included in future cohort studies.

Some participants gave feedback on the diagnosis options in the logbook. According to their findings, a significant number of patients experience mixed headaches, prompting the inclusion of this option in the logbook, as well as the following two questions to the survey: “Of the patients you see, what percentage would you estimate experience mixed headaches?” and “Which combination do you find most often?”. An overview of the domains in the final Danish Headache Questionnaire (DHQ) compared to the Australian version is outlined in Table 2. A complete list of additions or divergence from the original questionnaire can be found in appendix A. The final version consists of 47 questions.

**Table 2:** Content of survey in Danish final version versus Australian version

Domains	Australian version	Danish final version
Practitioner demographics	✓	✓
Headache prevalence	✓	*
Headache classification	✓	✓
Monitoring measures	✓	✓
Multidisciplinary care	✓	✓
Headache management	✓	✓
Knowledge of Danish guideline		✓
Mixed headaches and other headaches		✓
Medical history		✓
Physical examination and x-ray		✓
Side effects		✓

*Data collection in a separate logbook

### Development of a generic version of the Danish headache questionnaire

This process involved two steps: a cross-cultural adaptation and translation of the final version of the survey and logbook into English and generating a non-profession-specific generic version.

Minimal alterations were made during the translation process, although two issues were discussed. One issue was the term ‘health care practitioner’, which was changed to ‘health professional’ - a more appropriate term. The other issue was how a patient perceives the headache: whether a patient ‘suffers from’ or ‘experiences’ a headache. The latter term was chosen because the group decided that ‘suffering’ conveyed a more serious perception of headache, whereas ‘experiencing’ seemed to be more neutral. See Appendix B and C for the English version.

In the generic version, all profession-specific terms and questions have been omitted, modified where relevant or alternative options suggested to be appropriate for other clinical settings. For instance, the question “Where did you obtain your chiropractic education?” was changed to “Where did you obtain your [name of profession] education?”. Another change was made regarding the response options for the question on treatment: ”How often do you use the following treatment options in managing headache patients?”. The response options were modified to include only a few and a comment provided with the following suggestion: “Insert or remove relevant treatment options in the table, e.g., acupuncture, nutritional advice, and so forth”. For a generic version of the DHQ, see appendix D.

## Discussion

We have developed a comprehensive standardised questionnaire (the Danish Headache Questionnaire, DHQ), to assess the management of patients with headaches in primary care. The DHQ is applicable across various primary care settings and covers all relevant domains, including systematic registration of headache prevalence, diagnostic procedures, management strategies, and treatment modalities. The DHQ can be used as an educational tool useful to guide a complete and standardised assessment among primary care clinicians.

Also, the DHQ could be used as a basis for cohort studies to evaluate clinicians’ knowledge of guidelines and diagnostic criteria, as well as their use of different management and treatment strategies for patients with headaches in primary care. Identifying gaps in knowledge may help optimise the management and treatment of headache patients in primary care. Furthermore, understanding how these patients are managed, could inform the development of items relevant to include in a future randomised controlled trial on the effectiveness of management and treatment in this setting. Such insights could assist healthcare professionals, researchers, and policymakers in gaining a deeper understanding of the healthcare requirements for individuals with headaches.

### The survey instrument

We are confident in the feasibility of the DHQ, as well as the content validity and the clarity of its wording. Our focus on relevant content and ensuring that the target population comprehends the questionnaire items during the development phase has led to robust content validity. This was realised through an iterative process that involved integrating standard questionnaire data with interview data using COSMIN (Consensus-based Standards for the selection of health Measurement Instruments) as the main methodological framework [16].

The literature search to identify studies using headache questionnaires relevant to primary care, included terminology relating to ‘chiropractic’, ‘headache’, ‘diagnosis’, and ´questionnaire´. The initial decision to focus on chiropractic care, may, in retrospect, have limited the questionnaires found and omitted questionnaires from other primary care settings. However, to our knowledge, no other questionnaires as comprehensive as the DHQ exist that are designed to investigate the management of patients with headaches in primary care in general.

We deliberately abstained from using more specific diagnostic categories in the questionnaire, such as episodic or chronic tension-type headache, episodic or chronic migraine, or migraine with or without aura symptoms. While these details could have provided us with more insights into the types of headaches seen in primary care, we considered their inclusion overly extensive with too many response options potentially leading to fewer registrations. Nonetheless, we integrated the diagnostic criteria from the International Classification of Headache Disorders in the questionnaire [13]. Unlike the Australian study, we provided the diagnostic criteria for the different diagnoses directly in the questionnaire, ensuring participants were promptly reminded of the criteria.

This questionnaire is merely providing descriptive data on how clinicians manage patients, and it is not meant to provide data on what is right or wrong but may shed a light on knowledge gaps that should be addressed. As an example, in an unpublished study using the DHQ among Danish chiropractors, themes emerged that prompted to self-reflection within the profession, leading to the development and offering of supplementary training for the chiropractors and the undergraduate students. Ensuring a more consistent approach to patients with headaches among different primary care clinicians, both within and between professions, could result in a more efficient and coherent pathway for the patients. This could potentially benefit both patients and society by reducing absenteeism from work and improving quality of life.

### The logbook for tracking the prevalence of consultations for headaches

The choice of prospective registration of headache frequency using a logbook makes prevalence estimates more accurate and valid compared to using retrospective data as recall bias is minimised [28]. Furthermore, we chose a study period of four weeks as opposed to the two weeks employed in the study by Moore et al. [14] as we believe this choice gives a more precise estimation of the prevalence. However, it is worth noting that the four-week period may result in a lower participation rate due to the daily registration burden, potentially resulting in more dropouts. On the other hand, two weeks is a short time for the participants to become fully acquainted with the study procedures. Additionally, relying solely on a two-week timeframe may skew the results by chance, simply due to lower or higher number of headache patients in those weeks.

It should be noted that the prevalence estimates are at consultation level and not at patient level, which would provide even more accurate estimates and the possibility of looking at the number of consultations, and if the registration period had been longer, the duration of each headache episode could have been defined. However, this was a decision by the research group to keep the administrative burden on the participants low, in order to ensure continued participation.

### Use of the Danish headache questionnaire

The final DHQ is available in a generic and chiropractic version (both Danish and English) on a website about questionnaires in musculoskeletal research [29]. A user’s guide is also available on the website, providing general information on the structure and intended use of the DHQ, as well as suggestions for possible modifications to the questions and response categories.

### Strengths and limitations

The strength of this study is the comprehensiveness of the DHQ, which covers all relevant domains and is specifically designed to assess primary care clinicians’ management of patients with headache. We believe that it has good feasibility and content validity and is applicable in different primary care settings. However, we recognise that primary care settings are organised differently in various countries, which could potentially challenge the use of the DHQ. In the Danish setting where data were collected, the average time spent per consultation was 15 min [30] and users found that this one-time survey and logbook took little time to complete and was therefore feasible in a busy working day. We recommend that future studies employ a pilot test of the DHQ when applied in a different primary care setting.

The search strings for the initial literature search were unfortunately not saved correctly, so we cannot provide a more detailed search string than the terms listed in the manuscript and therefore cannot say whether the search was comprehensive enough, which is a limitation.

Additional items were included in the questionnaire to address domains not covered in the Australian version of the questionnaire. The included items were based on face validity and based on pilot testing in a Danish healthcare setting. As a result, the Danish version of the DHQ is tailored to the Danish healthcare context. However, each item in the English version was carefully reviewed and adapted, where necessary, to ensure broader applicability. We acknowledge the limitations of this approach, particularly given the research team’s limited experience with international healthcare systems. Instead, we followed the COSMIN methodology for assessing content validity, which is considered the most critical measurement property for this type of instrument. Although we did not include a formal qualitative study, as this was not considered necessary in our approach, we used a methodology that incorporated a thorough review of the existing headache literature and clinical guidelines, as well as extensive pilot testing. Although the absence of a qualitative study could be perceived as a limitation, we believe that our methodology adequately addressed content validity.

The cross-cultural adaptation followed a modified version of the process by Beaton et al., which is a limitation. In stage 4, we did not have all the translators a s part of the expert group and we were not able to perform a pilot test in a Danish setting, which should be incorporated in future studies.

## Conclusion

The Danish Headache Questionnaire has good content validity and is a feasible tool for measuring clinician characteristics and knowledge when managing patients with headaches and obtaining prevalence estimates in Danish chiropractic care and has the potential to be a useful tool for primary care in general. Furthermore, it can be used as an educational tool useful to guide a complete and standardised assessment. The generic version could be used in diverse primary care settings, ensuring a more standardised approach and facilitating comparisons between settings.

These findings suggest that the Danish Headache Questionnaire may be a valuable instrument for clinical assessment in primary care and have the potential to fill in gaps of knowledge which could improve the management of headache disorders in primary care. Furthermore, future studies could use the knowledge gained in randomised controlled trials on the effectiveness of management and treatment in primary care. This could give a deeper understanding of the most beneficial management and treatment strategy for individuals suffering from headaches.

## Electronic supplementary material

Below is the link to the electronic supplementary material.

Supplementary Material 1

Supplementary Material 2

Supplementary Material 3

Supplementary Material 4

Supplementary Material 5

Supplementary Material 6

## Data Availability

No datasets were generated or analysed during the current study.

## Abbreviations

DHQ: The Danish Headache Questionnaire
ICHD-3: International Classification of Headache Disorders
COSMIN: Consensus-based Standards for the selection of health Measurement Instruments
