# Supplementary material for: Development and preliminary validation of the Danish headache questionnaire
Source: Chiropr Man Therap. 2025 Feb 27;33:10. doi: 10.1186/s12998-025-00573-4 (PMC11866720; doi:10.1186/s12998-025-00573-4)
Supplement: Supplementary file 1 — Supplementary Material 1 [file 12998_2025_573_MOESM1_ESM.docx]

**LOGBOOK**

**Daily registration of patients with headaches**

Dear [name or profession]

Thank you for taking the time and willingness to participate in the headache project. In this logbook document, please record the following information for each headache patient you attend to during the day throughout the project period. Please record over 20 working days, equivalent to 4 weeks, considering any holidays, sickness, and part-time work.

How to fill out the form:

Fill in the date when the patient was seen, along with their date of birth, to make it easier for you to go back and complete the rest if it's not done immediately after the consultation.

In the rest, simply mark the relevant fields for today's consultation (see the example in the first line). It is recommended to record daily during the project period but do what suits your daily routine best. It may be advantageous to have the [relevant headache guideline and/or headache classification] lying next to you.

A **new patient** is defined as a consultation with a completely new headache patient who has not been seen in the clinic before.

A **new problem** is defined as a consultation with a patient who has been seen in the clinic before, but where headache is diagnosed as a new problem or a recurring issue.

A **follow-up** is defined as a follow-up consultation with a patient in an existing course of treatment for headache.

A **maintenance** is defined as a consultation where the patient is seen as needed and/or for preventive measures for headaches and is therefore not in a course of treatment.

If the headache is the **primary** reason for the visit, mark the primary cause.

If the headache is a **secondary** complaint to e.g. neck/shoulder pain, mark the secondary cause.

Please also specify the type of headache the patient is being treated for, i.e., what diagnosis has been treated on the day of the consultation.Øverst på formularen

Example of registration of patients with headaches

| **Date** | **Birthdate** | **New patient** | **New problem** | **Follow-up** | **Maintenance** | **Primary reason** | **Secondary reason** | **Migraine** | **Tension type** | **Cervicogenic** | **Other** |
| --- | --- | --- | --- | --- | --- | --- | --- | --- | --- | --- | --- |
|  |  |  |  |  |  |  |  |  |  |  |  |
|  |  |  |  |  |  |  |  |  |  |  |  |
|  |  |  |  |  |  |  |  |  |  |  |  |

**Daily registration of total number of patients**

Your registration of patients with headaches in the logbook is used for further frequency calculations. To do so, we need the total number of patients you see each day. In this form, please indicate all patients seen on the day, regardless of the reason for the visit, i.e., patients both with and without headaches.

A **new patient** is defined as a consultation with a completely new headache patient who has not been seen in the clinic before.

A **new problem** is defined as a consultation with a patient who has been seen in the clinic before, but where headache is diagnosed as a new problem or a recurring issue.

A **follow-up** is defined as a follow-up consultation with a patient in an existing course of treatment for headache.

A **maintenance** is defined as a consultation where the patient is seen as needed and/or for preventive measures for headaches and is therefore not in a course of treatment.

How to fill out the form:

The total number of patients within each category is recorded in the form for each working day. It may facilitate the process to have a notepad available and make marks throughout the day, making it quick to count and transfer the numbers at the end. It is recommended to record daily during the project period but do what suits your daily routine best. Please record over 20 working days, which do not necessarily have to be consecutive.

Example of registration of total number of patients

| **Day*** | **Date** | **New patient** | **New problem** | **Follow-up** | **Maintenance** |
| --- | --- | --- | --- | --- | --- |
| 1 |  |  |  |  |  |
| 2 |  |  |  |  |  |
| 3 |  |  |  |  |  |
| 4 |  |  |  |  |  |

*Number day of the 20-day registration period
