# Supplementary material for: Development and preliminary validation of the Danish headache questionnaire
Source: Chiropr Man Therap. 2025 Feb 27;33:10. doi: 10.1186/s12998-025-00573-4 (PMC11866720; doi:10.1186/s12998-025-00573-4)
Supplement: Supplementary file 3 — Supplementary Material 3 [file 12998_2025_573_MOESM3_ESM.docx]

| Deviations based on feedback from participants in the pilot studies as well as debate within the research group. |
| --- |
| Numbers in parentheses are the corresponding question numbers in the ACORN questionnaire. |

| Question # in final DK version | Question # in first DK version | Danish version 1 | Original ACORN | Addition/deviation from ACORN | Addition/deviation between DK version 1 and final version | Translation of supplemental question |
| --- | --- | --- | --- | --- | --- | --- |
|  |  | Demography | Practitioner demographichs | None |  |  |
| #1 | #1 | Hvad er dit køn | What is your gender? (1) | None | "other" is changed to "other gender identity" |  |
| #2 | #2 | Hvad er din alder? |  | Question added to gain more knowledge of the participants |  |  |
| #2a | #2a | Hvor mange dage om ugen arbejder du i klinik? |  |  | Added to take into account those who work part time or is on vacation and thus be able to find the real prevalence when combining data with the logbook | How many days a week do you work in the clinic? |
| #3 | #3 | Hvor mange år har du været aktiv som kliniker? (turnus ikke medregnet) | How many years have you been in practice? (2) | Specification of postgraduate year not in DK version | Postgrauate year added as answer option, since we believe they have a good memory on the diagnostic criteria and should be included |  |
| #4 | #4 | Hvor har du modtaget din uddannelse som kiropraktor? | Please identify where you received your chiropractic education (3) | None | Answer options downgraded to DK, US, UK, Canada, other |  |
| #5 |  | Antal kiropraktorer i klinikken på din primære arbejdsplads (inkl. dig selv): |  |  | Added to gain more knowledge about the clinics | Number of chiropractors in your primary place of occupation |
| #6 |  | Øvrige fagpersoner i klinikken (sættes kryds hvis ja; flere svar muligt) |  |  | Added to gain more knowledge on multidisciplinary management | Other health care practitioners at the clinic? (Cross off all relevant answers) |
| #7 | #5 | Har du andre uddannelser? |  | Question added to gain more knowledge of the participants |  |  |
| #8 | #6 | I hvilken region praktiserer du? (primære arbejdsplads) | Where do you currently practice? (You may select more than one response) (4) | In the DK version you can choose one region |  |  |
|  |  | Hovedpine prævalens | Headache prevalence |  |  |  |
|  | #7 | Hvor mange nye patienter har du tilset? (alle henvendelsesårsager) | In the last two weeks, how many new patients did you consult? (5) | In the DK version, the duration of registration is not included in the question, but in the corresponding text | The whole prevalence section #7-15 has been withdrawn due to feedback from pilot test participants. It was too time consuming and the summing up of patients from the logbook has now been transferred to the research group |  |
|  | #8 | Hvor mange nye patienter havde hovedpine som primær henvendelsesårsag? | In the last two weeks, how many new patients did you consult who had a chief complaint of headache? (6) | AM | AM |  |
|  | #9 | Hvor mange nye patienter havde hovedpine som ledsagesymptomer til anden primær henvendelsesårsag? | In the last two weeks, how many new patients did you consult who had a secondary complaint of headache (i.e. headache present but not their chief complaint)? (7) | AM | AM |  |
|  | #10 | Hvor mange eksisterende patienter har du tilset? (alle henvendelsesårsager) |  | Question added to take all patients with headache into account and not only new patients, and thereby estimate the total magnitude of patients with headache | AM | How many patients already known in the clinic, did you consult? |
|  | #11 | Hvor mange eksisterende patienter havde hovedpine som primær henvendelsesårsag? |  | AM | AM | How many new patients already known in the clinic, did you consult who had a chief complaint of headache? |
|  | #12 | Hvor mange eksisterende patienter havde hovedpine som ledsagesymptomer til anden primær henvendelsesårsag? |  | AM | AM | How many patients already known in the clinic, did you consult who had a secondary complaint of headache? |
|  | #13 | Hvor mange konsultationer har du haft i alt (Alle henvendelsesårsager) | In the last two weeks, how many new patients did you consult? (5) | It is specified in the DK version that the question includes all causes of consult | AM |  |
|  | #14 | I hvor mange af disse konsultationer, var hovedpine den primære henvendelsesårsag? | In the last two weeks, how many consultations did you undertake where the chief complaint was headache? (9) | In the DK version, the duration of registration is not included in the question, but in the corresponding text | AM |  |
|  | #15 | I hvor mange af disse konsultationer, var hovedpine et ledsagesymptom til anden primær henvendelsesårsag? | In the last two weeks, how many consultations did you undertake where the secondary complaint was headache (i.e headache present but not the chief complaint)? (10) | AM | AM |  |
|  |  | Hovedpinevejledning fra DSK | Headache guideline DCS | Section added to gain more knowledge on the familiarity and use of the Danish clinical guideline |  |  |
| #9 | #16 | Kender du indholdet i hovedpinevejledningen fra DSK? |  | Question added |  | Are you familiar with the contents of the headache guidelines from DCS? |
| #10 | #17 | Har vejledningen ændret din praksis? |  | Question added |  | Have these guidelines changed the way you practice daily? |
| #11 | #17.1 | Hvis ja, inden for hvilke områder? |  | Question added |  | In which areas? (Indicate yes/no where relevant) |
|  |  | Primære hovedpinetyper | Headache classification | Questions on primary, secondary and other types of hedache are separated in the DK version |  |  |
| #12 | #18 | Er du bekendt med nedenstående diagnostiske kriterier for spændingshovedpine? | Are you familiar with these diagnostic criteria for these primary headaches? (11) | In the ACORN there is a short resume of the criteria for the primary (tension-type, migraine and cluster headache) and the secondary (cervicogenic and medicine overuse headache), and there is only one overall question for primary and secondary types. In the DK version all diagnostic criteria are shown for the 5 mentioned types and the question is asked for each type. This splitup was chosen, since it seems impossible to say yes to this question, because you may be familliar with 2/3 diagnoses. It was also due to the discussion in the Australian manuscript, to make it more clear to the reader. | Word change: the Danish word for "familiar" was changed to another more appropriate word, which seemed more exhaustive. "Bekendt med" changed to "fortrolig med". |  |
|  |  | Angiv hvilke elementer i de diagnostiske kriterier for spændingshovedpine, du er *moderat eller mindre*fortrolig med (hvis du er meget fortrolig med kriterierne, kan du gå videre til næste side): |  |  | Added after feedback from pilot test participants. Will give more knowledge on which specific elements in the criteria are mostly known or not. It is possible to tick off each element | Indicate with which elements of the diagnostic criteria for tension-type headaches you are either moderately or slightly familiar. (If you are very familiar with these criteria, please continue to the next page.) |
| #13 | #19 | Er du bekendt med nedenstående diagnostiske kriterier for migræne? |  | AM | Word change: the Danish word for "familiar" was changed to another more appropriate word, which seemed more exhaustive. "Bekendt med" changed to "fortrolig med". |  |
|  |  | Angiv hvilke elementer i de diagnostiske kriterier for migræne, du er *moderat eller mindre*fortrolig med (hvis du er meget fortrolig med kriterierne, kan du gå videre til næste side): |  |  | Added after feedback from pilot test participants. Will give more knowledge on which specific elements in the criteria are mostly known or not. It is possible to tick off each element | Indicate with which elements of the diagnostic criteria for migraine you are either moderately or slightly familiar. (If you are very familiar with these criteria, please continue to the next page.) |
| #14 | #20 | Er du bekendt med nedenstående diagnostiske kriterier for klyngehovedpine? |  | AM | Word change: the Danish word for "familiar" was changed to another more appropriate word, which seemed more exhaustive. "Bekendt med" changed to "fortrolig med". |  |
|  |  | Angiv hvilke elementer i de diagnostiske kriterier for klyngehovedpine, du er *moderat eller mindre*fortrolig med (hvis du er meget fortrolig med kriterierne, kan du gå videre til næste side): |  |  | Added after feedback from pilot test participants. Will give more knowledge on which specific elements in the criteria are mostly known or not. It is possible to tick off each element | Indicate with which elements of the diagnostic criteria for cluster headaches you are either moderately or slightly familiar. (If you are very familiar with these criteria, please continue to the next page.) |
| #15 | #21 | Tager din diagnose udgangspunkt i de diagnostiske kriterier for primære hovedpinetyper? | Do you use these diagnostic criteria for primary headache types such as migraine, tension-type headache or cluster headache? (12) | Not specified in DK version |  |  |
| #16 | #22 | I hvilket omfang er du enig i følgende udsagn angående de diagnostiske kriterier for primære hovedpinetyper? | To what extent do you agree with the following statements about these criteria for the diagnosis of primary headaches. (13) | Option 5 + 6 in ACORN is combined into one option in the DK version, which we thought covered the information needed. In the DK version the answer options are supplemented with smiley-faces | The smiley faces are taken out. Studies have shown that it may introduce bias, because participants are more likely to choose the happy smiley |  |
|  |  | Sekundære hovedpinetyper | Headache Classification | Questions on primary, secondary and other types of hedache are separated in the DK version |  |  |
| #17 | #23 | Er du bekendt med nedenstående diagnostiske kriterier for cervicogen hovedpine? | Are you familiar with these diagnostic criteria for these secondary headaches? (14) | This splitup was chosen, since it seems impossible to say yes to this question, because you may be familliar with 2/3 diagnoses. It was also due to the discussion in the Australian manuscript, to make it more clear to the reader. |  |  |
|  |  | Angiv hvilke elementer i de diagnostiske kriterier for cervicogen hovedpine, du er *moderat eller mindre*fortrolig med (hvis du er meget fortrolig med kriterierne, kan du gå videre til næste side): |  |  | Added after feedback from pilot test participants. Will give more knowledge on which specific elements in the criteria are mostly known or not. It is possible to tick off each element | Indicate with which elements of the diagnostic criteria for cervicogenic headaches you are either moderately or slightly familiar. (If you are very familiar with these criteria, please continue to the next page.) |
| #18 | #24 | Er du bekendt med nedenstående diagnostiske kriterier for medicinoverforbrugshovedpine? |  | This splitup was chosen, since it seems impossible to say yes to this question, because you may be familliar with 2/3 diagnoses. It was also due to the discussion in the Australian manuscript, to make it more clear to the reader. |  |  |
|  |  | Angiv hvilke elementer i de diagnostiske kriterier for medicinoverforbrugshovedpine, du er *moderat eller mindre*fortrolig med (hvis du er meget fortrolig med kriterierne, kan du gå videre til næste side): |  |  | Added after feedback from pilot test participants. Will give more knowledge on which specific elements in the criteria are mostly known or not. It is possible to tick off each element | Indicate with which elements of the diagnostic criteria for medication-overuse headaches you are either moderately or slightly familiar. (If you are very familiar with these criteria, please continue to the next page.) |
| #19 | #25 | Tager din diagnose udgangspunkt i de diagnostiske kriterier for sekundære hovedpinetyper? | Do you use these diagnostic criteria for secondary headache types such as cervicogenic or medication overuse headache? (15) | Not specified in DK version |  |  |
| #20 | #26 | I hvilket omfang er du enig i følgende udsagn angående de diagnostiske kriterier for sekundære hovedpinetyper? | To what extent do you agree with the following statements about these headache diagnostic criteria for the diagnosis of secondary headaches. (16) | Option 5 + 6 in ACORN is combined into one option in the DK version, which we thought covered the information needed. In the DK version the answer options are supplemented with smiley-faces | The smiley faces are taken out. Studies have shown that it may introduce bias, because participants are more likely to choose the happy smiley |  |
|  |  | Andre typer | Headache classification | Questions on primary, secondary and other types of hedache are separated in the DK version |  |  |
| #21 | #27 | Anvender du andre betegnelser eller ord til at diagnosticere hovedpinetyper, foruden dem der er anbefalet af DSK og ICHD-klassifikationen? (primær eller sekundær) | Do you use other labels or words to diagnose headache types other than those recommended by the ICHD classification (primary or secondary)? (17) | DCS added |  |  |
| #21b |  | Hvor mange procent af de hovedpinepatienter, du ser, vil du vurdere har blandingshovedpine? |  |  | Added to gain more knowledge on mixed headaches, since this is experienced by many patients according to the pilot test participants | Of the patients you see, what percentage would you estimate suffer from combination headaches? |
| #21c |  | Hvilken kombination ser du hyppigst? |  |  | AM | Which combination of headaches do you find most often? |
|  |  | Anamnese & Undersøgelse |  | Section added to gain more knowledge on management of patients with headache |  |  |
| #22 | #28 | Hvor ofte indeholder din anamnese nedenstående elementer i forbindelse med udredning og diagnostik af nye hovedpinepatienter? |  | Question added to gain more knowledge on whats included in the medical history. Answer options are according to the Danish guideline |  | How often does your medical history contain the following elements, as a part of the evaluation and diagnosis of new headache patients? |
| #23 |  | Hvor ofte foretager du røntgen optagelse af columna hos patienter med følgende hovedpinetyper? |  |  | Question added to gain knowledge on how often x-ray is being used | How often do you conduct an x-ray examination of a patient's columna, in connection with the following types of headaches? |
| #24 | #29 | Hvor ofte indeholder din objektive undersøgelse nedenstående elementer i forbindelse med udredning og diagnostik af nye hovedpinepatienter? |  | Question added to gain more knowledge on whats included in the examination. Answer options are according to the Danish guideline |  | How often does your objective examination contain the following elements, as a part of the investigation and diagnosis of new headache patients? |
|  |  | Monitorering & Behandlingseffekt | Treatment outcome measures | None |  |  |
| #25 | #30 | Hvor ofte benytter du følgende redskaber til monitorering af patienter med hovedpine som den primære henvendelsesårsag? | How often do you use the following outcome measures to monitor new patients who present with a chief complaint of headache? (19) | In the DK version, the answer options are changed to "headache diary" and "headache calendar" |  |  |
|  | #31 | Benytter du andre redskaber til monitorering af patienter med hovedpine som den primære henvendelsesårsag? |  | Answer options from ACORN (HDI and MIDAS) are transferred to this question in the DK version | This question is removed in this version, since very few use these tools and to shorten the questionnaire.It is therefore seen as included within "other" in the previous question, where there is an option to write which other is used |  |
|  |  | Tværfagligt Samarbejde | Multidisciplinary Care | None |  |  |
| #26 | #32 | Hvor ofte modtager du patienthenvisninger/anbefalinger fra følgende sundhedsprofessionelle i forbindelse med håndtering af hovedpinepatienter? | How often do you receive a patient referral from the following healthcare professionals for the management of headache? (20) | In the DK version #referral" is translated to "referral/recommendation", since this suits the Danish healthcare system better. In ACORN "osteopath" is a separate answer option, in the DK version it goes under "other". "CAM practitioner" is changed in the DK version to "other caretaker". "Massage therapist" becomes a separate option. "Health visitor/district nurse" is added as option. In the DK version an "always" answer option is added. |  |  |
| #27 | #33 | Hvor ofte henviser du patienter til følgende sundhedsprofessionelle i forbindelse med håndtering af hovedpinepatienter? | How often do you refer a patient to the following healthcare professionals for the management of headache? (21) | In ACORN "osteopath" is a separate answer option, in the DK version it goes under "other". "CAM practitioner" is changed in the DK version to "other caretaker". "Massage therapist" becomes a separate option. "Health visitor/district nurse" is added as option. In the DK version an "always" answer option is added. |  |  |
| #28 | #34 | Røde flag: I hvilket omfang er du bekendt med følgende indikationer for yderligere (akut) udredning hos patienter med hovedpine? | To what extent are you aware of the following indications for urgent medical attention in patients with headache (red flags)? (23) | The DK version is changed according to the DCS guideline. |  |  |
| "29 | #35 | Hvor ofte vil du henvise patienter med hovedpine til andre sundhedsprofessionelle, baseret på følgende årsager? | How often would you refer a patient with headache to another healthcare professional based on the following reasons? (22) | Answer option "sometimes" is added to DK version |  |  |
|  |  | Hovedpinehåndtering I | Chiropractic Headache management | This section is split into two sections in the DK version |  |  |
| #30 | #36 | Hvor vigtige er følgende behandlingsresultater i din håndtering af hovedpinepatienter? | How important are the following treatment outcomes to your management of patients with headache? (24) | None |  |  |
| #31 | #38 | Spændingshovedpine: Hvor ofte bruger du følgende behandlingsmuligheder i din håndtering af hovedpinepatienter? | How frequently do you use the following treatment options in your management of patients with tension headache? (26) | Categories changed: "Non-thrust spinal manipulaton" changed to "mobilisation without impulse", "instrument adjusting" changed to "activator". Addition of "toggle recoil". "Stretching" removed in DK version, is included in "Exercises for neck/shoulder". Addition of "dry needling/acupuncture". ”Soft tissue or exercise therapy to temporo- mandibular region” is simplified to "Jaw treatment". "Advice on diet or fitness" is split into two: "Advice on diet" and "advice on fitness". Response option "sometimes" is added. |  |  |
| #32 | #37 | Migræne: Hvor ofte bruger du følgende behandlingsmuligheder i din håndtering af hovedpinepatienter? | How frequently do you use the following treatment options in your management of patients with migraine? (25) | Categories changed: "Non-thrust spinal manipulaton" changed to "mobilisation without impulse", "instrument adjusting" changed to "activator". Addition of "toggle recoil". "Stretching" removed in DK version, is included in "Exercises for neck/shoulder". Addition of "dry needling/acupuncture". ”Soft tissue or exercise therapy to temporo- mandibular region” is simplified to "Jaw treatment". "Advice on diet or fitness" is split into two: "Advice on diet" and "advice on fitness". Response option "sometimes" is added. |  |  |
| #33 | #39 | Cervicogen hovedpine: Hvor ofte bruger du følgende behandlingsmuligheder i din håndtering af hovedpinepatienter? | How frequently do you use the following treatment options in your management of patients with cervicogenic headache? (27) | Categories changed: "Non-thrust spinal manipulaton" changed to "mobilisation without impulse", "instrument adjusting" changed to "activator". Addition of "toggle recoil". "Stretching" removed in DK version, is included in "Exercises for neck/shoulder". Addition of "dry needling/acupuncture". ”Soft tissue or exercise therapy to temporo- mandibular region” is simplified to "Jaw treatment". "Advice on diet or fitness" is split into two: "Advice on diet" and "advice on fitness". Response option "sometimes" is added. |  |  |
|  |  | Hovedpinehåndtering II | Chiropractic Headache management | This section is split into two sections in the DK version |  |  |
| #34 | #40 | Angiv det gennemsnitlige antal konsultationer du forventer i behandlingen af en ny patient med følgende hovedpinetyper som primære klage. | Indicate the average number of visits you provide during the initial period of care for a new patient presenting with a chief complaint of headache as listed below. (28) | Change of response options in the DK version: (“Less than 5 treatments”, “5-10 treatments”, ”More than 10 treatments”) to ("Under 5”, ”5-7”, ”8-10”, ”More than 10”). ACORN uses ”the initial period”. This is left out in the DK version, since this is believed to be implicit. | Change of response options to "0-4", "5-7", "8-10", "11 or more", since this seemed more precise. We decided to skip the division in #less than/more than 3 months duration" for all 3 types of headache due to feedback from pilot participants and to make it more simple |  |
| #35 | #41 | I den indledende fase: Angiv den forventede gennemsnitlige varighed i behandlingen af en ny patient med følgende hovedpinetyper som primære klage. | Indicate the average duration of the initial period of care for a new patient presenting with a chief complaint of headache as listed below. (29) | Change of response options in the DK version: (Less than 2 weeks", "2-4 weeks", "4-8 weeks", "More than 8 weeks") to ("1-2 weeks", "3-5 weeks", "6-8 weeks", "9 weeks or more". Change due to feedback from preliminary test persons. In ACORN, there is no differentation between primary headache types, this is added in DK version. | Change of question: initial period was removed, since this confused the participants and it is difficult to determine how long the initial period is. In "new patient" it is believed to be implicit that it is the initial period. We decided to skip the division in #less than/more than 3 months duration" for all 3 types of headache due to feedback from pilot participants and to make it more simple |  |
| #36 | #42 | I den indledende fase: Angiv den forventede gennemsnitlige hyppighed i behandlingen af en ny patient med følgende hovedpinetyper som primære klage. | Indicate the average frequency of visits for the initial period of care for a new patient presenting with a chief complaint of headache as listed below. (30) | In ACORN, there is no differentation between primary headache types, this is added in DK version. | Change of question: initial period was removed, since this confused the participants and it is difficult to determine how long the initial period is. In "new patient" it is believed to be implicit that it is the initial period. We decided to skip the division in #less than/more than 3 months duration" for all 3 types of headache due to feedback from pilot participants and to make it more simple |  |
|  |  | Hovedpinehåndtering III | Chiropractic Headache management | This section is split into two sections in the DK version |  |  |
| #37 | #43 | Hvor effektiv opfatter du den behandling du som kiropraktor giver af følgende hovedpinetyper? | How effective do you perceive your chiropractic management to be for each of the following headachetypes? (31) | None | We decided to skip the division in #less than/more than 3 months duration" for all 3 types of headache due to feedback from pilot participants and to make it more simple |  |
| #38 |  | Beskriv de hyppigst oplevede bivirkninger efter behandling af patienter med hovedpine |  |  | Question added to gain knowledge on which side-effects are experienced. This was chosen due to the discussion in the original Australian manuscript and to give information on which questons should be asked in a potential other cohort study | Describe the most commonly experienced side-effects after treatment of patients with headaches |
