# Supplementary material for: Development and preliminary validation of the Danish headache questionnaire
Source: Chiropr Man Therap. 2025 Feb 27;33:10. doi: 10.1186/s12998-025-00573-4 (PMC11866720; doi:10.1186/s12998-025-00573-4)
Supplement: Supplementary file 4 — Supplementary Material 4 [file 12998_2025_573_MOESM4_ESM.docx]

**“The Danish Headache Questionnaire on management and treatment of headache in chiropractic practice”**

**English version of the survey for chiropractors**

| **Question #** | **English version** |
| --- | --- |
|  | 1. **Demography**   This section concerns you as a clinician |
| 1 | What is your gender?   1. Female 2. Male 3. Other gender identity |
| 2 | How old are you? |
| 3 | How many days per week do you work in the clinic?   1. One 2. Two 3. Three 4. Four 5. Five (+) |
| 4 | How many years have you been actively practicing?   1. Postgraduate internship 2. 1-4 3. 5-10 4. 11-15 5. >15 |
| 5 | Where did you obtain you chiropractic education?   1. Denmark 2. The UK 3. The US 4. Canada 5. Other - please state |
| 6 | Including yourself, how many chiropractors are there at your primary workplace? |
| 7 | Are there other health professionals at the clinic?  (Cross off all relevant answers)   1. Medical doctor 2. Physiotherapist 3. Massage therapist 4. Acupuncturist 5. Other health professionals (please state) |
| 8 | Do you have other educations?   1. No 2. Yes 3. If yes, which educations? |
| 9 | In which Region do you have your primary workplace?   1. North Jutland 2. Mid Jutland 3. Southern Denmark 4. Zealand 5. Copenhagen 6. Outside Denmark's borders |
|  | 1. **Headache Guideline from the Danish Chiropractic Society (DCS)**   This section concerns your knowledge and application of the headache guideline from DCS (2019). |
| 10 | Are you familiar with the contents of the headache guideline from DCS?   1. Yes, all of it 2. Most of it 3. Parts of it 4. No |
| 11 | Has the guideline changed the way you practice daily?   1. Yes, a lot 2. Yes, to a certain extent 3. No, I was already following the guidelines 4. No, not at all   If yes, continue to question 11, if not continue to question 12. |
| 12 | In which areas? (Indicate yes/no where relevant)   1. Medical history 2. Objective examination 3. Diagnosis 4. Treatment 5. Referral 6. Another area |
|  | 1. **Primary types of headaches.**   This section concerns the use of diagnostic criteria for the primary headache types.  Questions 13-20 concerns the diagnostic criteria for primary types of headaches as recommended by DCS according to the International Classification of Headache Disorders (ICHD). |
| 13 | How familiar are you with the following diagnostic criteria for tension-type headaches?   1. Very familiar 2. Moderately familiar 3. Slightly familiar 4. Not at all familiar |
| 14 | Indicate with which parts of the diagnostic criteria for tension-type headaches you are either *moderately familiar, slightly familiar, or not at all familiar* with.  Cross off all relevant answers.  If you are very familiar with these criteria, please continue to question 15.  (Diagnostic criteria inserted here) |
| 15 | How familiar are you with the following diagnostic criteria for migraine headaches?   1. Very familiar 2. Moderately familiar 3. Slightly familiar 4. Not at all familiar |
| 16 | Indicate with which parts of the diagnostic criteria for migraine headaches that you are *either moderately familiar, slightly familiar, or not at all familiar* with. Cross off all relevant answers.  If you are very familiar with these criteria, please continue to question 17.  (Diagnostic criteria inserted here) |
| 17 | How familiar are you with the following diagnostic criteria for cluster headaches?   1. Very familiar 2. Moderately familiar 3. Slightly familiar 4. Not at all familiar |
| 18 | Indicate with which parts of the diagnostic criteria for cluster headaches that you are either *moderately familiar, slightly familiar, or not at all familiar* with.  Cross off all relevant answers.  If you are very familiar with these criteria, please continue to question 19. |
| 19 | Is your diagnosis for types of primary headaches based on the diagnostic criteria?   1. Yes 2. No |
| 20 | To what extent do you agree with the following statements regarding the diagnostic criteria of primary types of headaches?   \|  \| Strongly disagree \| Disagree \| Neither agree nor disagree \| Agree \| Strongly agree \| \| --- \| --- \| --- \| --- \| --- \| --- \| \| The criteria for diagnosing primary headaches are clear \|  \|  \|  \|  \|  \| \| The criteria of primary headache are easy to apply \|  \|  \|  \|  \|  \| \| The criteria fit well with my patients with primary headache \|  \|  \|  \|  \|  \| \| The diagnostic criteria have an influence on my management of patients with primary headache types \|  \|  \|  \|  \|  \| \| The diagnostic criteria help me to communicate with other healthcare professionals. (e.g., patient referrals and interdisciplinary treatment) \|  \|  \|  \|  \|  \| |
|  | 1. **Secondary types of headaches.**   This section concerns the use of diagnostic criteria for secondary types of headaches.  Questions 21-26 concerns the diagnostic criteria for secondary types of headaches as recommended by DCS according to the International Classification of Headache Disorders (ICHD). |
| 21 | How familiar are you with the following diagnostic criteria for cervicogenic headaches?   1. Very familiar 2. Moderately familiar 3. Slightly familiar 4. Not at all familiar |
| 22 | Indicate with which parts of the diagnostic criteria for cervicogenic headaches that you are either *moderately familiar, slightly familiar, or not at all familiar* with. Cross off all relevant answers.  If you are very familiar with these criteria, please continue to question 23.  (Diagnostic criteria inserted here). |
| 23 | How familiar are you with the following diagnostic criteria for medication overuse headaches?   1. Very familiar 2. Moderately familiar 3. Slightly familiar 4. Not at all familiar |
| 24 | Indicate with which parts of the diagnostic criteria for medication overuse headaches that you are either *moderately familiar, slightly familiar, or not at all familiar* with. Cross off all relevant answers.  If you are very familiar with these criteria, please continue to question 25.  (Diagnostic criteria inserted here) |
| 25 | Is your diagnosis for secondary types of headaches based on the diagnostic criteria?   1. Yes 2. No |
| 26 | To what extent do you agree with the following statements concerning the diagnostic criteria for secondary types of headaches?   \|  \| Strongly disagree \| Disagree \| Neither agree nor disagree \| Agree \| Strongly agree \| \| --- \| --- \| --- \| --- \| --- \| --- \| \| The criteria for diagnosing secondary headaches are clear \|  \|  \|  \|  \|  \| \| The criteria of secondary headache are easy to apply \|  \|  \|  \|  \|  \| \| The criteria fit well with my patients with secondary headache \|  \|  \|  \|  \|  \| \| The diagnostic criteria have an influence on my management of patients with secondary headache types \|  \|  \|  \|  \|  \| \| The diagnostic criteria help me to communicate with other healthcare professionals. (e.g., patient referrals and interdisciplinary treatment) \|  \|  \|  \|  \|  \| |
|  | **5. Other types of headaches and combination headaches.** |
| 27 | Do you use other terms to diagnose types of headaches in addition to those recommended by the DCS and ICHD classification (primary or secondary)?   1. Yes 2. No 3. If yes, which other terms do you use? (Please state) |
| 28 | Of the patients you see, what percentage would you estimate experience combination headaches?   1. 0-10% 2. 11-20% 3. 21-30% 4. 31-50% 5. >50% |
| 29 | Which combination do you find most often?   1. Migraine + tension-type 2. Migraine + cervicogenic 3. Cervicogenic + tension-type 4. Other combinations |
|  | 1. **Medical history and physical examination**   This section concerns your method of medical history and physical examination of headache patients.  Medical history and physical examination can discern primary headache types from secondary, by uncovering any warning signs of a serious secondary headache. It should show if the patient has other physical or mental health complications, which could be significant to the headache. |
| 30 | How often does the medical history contain the following elements, as a part of the evaluation and diagnosis of new headache patients?  (A 'new patient' is defined as a patient who has never been to the clinic before, a current patient presenting with headaches as a new condition or a current patient with recurring headache after at least six months without headache.)   \|  \| Never \| Rarely \| Sometimes \| Often \| Always \| \| --- \| --- \| --- \| --- \| --- \| --- \| \| General medical history (general condition of health, previous trauma etc.) \|  \|  \|  \|  \|  \| \| Description of the headache (localization, debut, duration, etc.) \|  \|  \|  \|  \|  \| \| The character of the headache (Intensity, quality of associated pain, accompanying symptoms, daily variation, etc.) \|  \|  \|  \|  \|  \| \| Causes (trigger factors, predisposing factors, aggravating or relieving factors, etc.) \|  \|  \|  \|  \|  \| \| Predisposing factors (neck and/or jaw pain, physical exertion, violent cough, stress, etc.) \|  \|  \|  \|  \|  \| \| Reaction pattern (Effect on level of function, medicine consumption) \|  \|  \|  \|  \|  \| \| General condition of health between episodes \|  \|  \|  \|  \|  \| \| Previous examinations and treatment \|  \|  \|  \|  \|  \| |
| 31 | How often do you carry out an x-ray examination of the spine in patients with the following types of headaches?   \|  \| Never \| Rarely \| Sometimes \| Often \| Always \| \| --- \| --- \| --- \| --- \| --- \| --- \| \| Tension-type headache \|  \|  \|  \|  \|  \| \| Migraine \|  \|  \|  \|  \|  \| \| Cervicogenic headache \|  \|  \|  \|  \|  \| |
| 32 | How often does your physical examination contain the following elements, as a part of the investigation and diagnosis of new headache patients?  (A 'new patient' is defined as a patient who has never been to the clinic before, a current patient presenting with headaches as a new condition or a current patient with recurring headache after at least six months without headache.)   \|  \| Never \| Rarely \| Sometimes \| Often \| Always \| \| --- \| --- \| --- \| --- \| --- \| --- \| \| Blood pressure measurement \|  \|  \|  \|  \|  \| \| Examination of the neck (ROM, soft tissue, and motion palpation) \|  \|  \|  \|  \|  \| \| Gait assessment \|  \|  \|  \|  \|  \| \| Evaluation of mental acuity. (e.g., relevant answers to questions) \|  \|  \|  \|  \|  \| \| Cranial nerve screening (pupil reaction, eye movement, sensory and motor function of the facial area, mouth and tongue function, soft palate function) \|  \|  \|  \|  \|  \| \| Neurological screening of the upper extremities, (trophic function, muscle tonus, muscle strength, sensory function, reflexes) \|  \|  \|  \|  \|  \| \| Coordination and balance (e.g., Romberg's test) \|  \|  \|  \|  \|  \| |
|  | 1. **Monitoring and treatment effectiveness**   This section concerns the tools you might use to monitor headache patients.  A **headache diary** can be helpful in finding a diagnosis and will therefore be useful in the early stages of a headache investigation. DCS recommends headache diaries in electronic or paper form, which can be found on the Migraine and Headache Association's website.  **A headache calendar** - can primarily be used for the follow up of headache treatment and DCS recommends the calendar available on Migraine and Headache Association's website. |
| 33 | How often do you use the following tools to monitor headache patients?   \|  \| Never \| Rarely \| Sometimes \| Often \| Always \| \| --- \| --- \| --- \| --- \| --- \| --- \| \| Headache diary \|  \|  \|  \|  \|  \| \| Headache calendar \|  \|  \|  \|  \|  \| \| Other \|  \|  \|  \|  \|  \|   If you use other tools for monitoring, please state which. |
|  | 1. **Interdisciplinary cooperation**   This section concerns your cooperation with other healthcare professionals in the management of headache patients. |
| 34 | How often do you receive referrals from the following health professionals in relation to the management of headache patients?   \|  \| Never \| Rarely \| Sometimes \| Often \| Always \| \| --- \| --- \| --- \| --- \| --- \| --- \| \| Medical doctor \|  \|  \|  \|  \|  \| \| Medical specialist (e.g., neurologist, rheumatologist, psychiatrist, etc.) \|  \|  \|  \|  \|  \| \| Psychologist \|  \|  \|  \|  \|  \| \| Dentist \|  \|  \|  \|  \|  \| \| Physiotherapist \|  \|  \|  \|  \|  \| \| Health visitor/district nurse \|  \|  \|  \|  \|  \| \| Massage therapist \|  \|  \|  \|  \|  \| \| Other therapist \|  \|  \|  \|  \|  \|   If you receive referrals from other therapists, please state which therapists. |
| 35 | How often do you refer/recommend patients to the following health professionals in connection with the management of headache patients?   \|  \| Never \| Rarely \| Sometimes \| Often \| Always \| \| --- \| --- \| --- \| --- \| --- \| --- \| \| Medical doctor \|  \|  \|  \|  \|  \| \| Medical specialist (e.g., neurologist, rheumatologist, psychiatrist, etc.) \|  \|  \|  \|  \|  \| \| Psychologist \|  \|  \|  \|  \|  \| \| Dentist \|  \|  \|  \|  \|  \| \| Physiotherapist \|  \|  \|  \|  \|  \| \| Health visitor/district nurse \|  \|  \|  \|  \|  \| \| Massage therapist \|  \|  \|  \|  \|  \| \| Other therapist \|  \|  \|  \|  \|  \|   If you refer patients to other therapists, please state which therapists. |
| 36 | Red flags: To what extent are you familiar with the following indications for further (acute) investigation in patients with headache?   \|  \| Not familiar \| Partly familiar \| Very familiar \| \| --- \| --- \| --- \| --- \| \| Thunderclap headache (sudden onset, intense headache) \|  \|  \|  \| \| Symptoms of increased intracranial pressure (morning headache, aggravation by coughing/sneezing/diaphragm pressure, impaired vision) \|  \|  \|  \| \| Newly occurring headache in a cancer or HIV patient \|  \|  \|  \| \| Headache/facial pain accompanied by fever or neurological deficits \|  \|  \|  \| \| Progressing headache over weeks \|  \|  \|  \| \| Newly occurring headache in patients under 10 or over 40 years of age \|  \|  \|  \| \| Nocturnal headaches in children \|  \|  \|  \| |
| 37 | How often do you refer patients with headache to other health professionals based on the following reasons?   \|  \| Never \| Rarely \| Sometimes \| Often \| Always \| \| --- \| --- \| --- \| --- \| --- \| --- \| \| To confirm/move closer to a diagnosis \|  \|  \|  \|  \|  \| \| To improve the patient's ability to self-manage headache-related loss of function \|  \|  \|  \|  \|  \| \| To investigate a ‘red flag’ (acute investigation) \|  \|  \|  \|  \|  \| \| For the purpose of pain relief treatment in a case of acute headache \|  \|  \|  \|  \|  \| \| For the purpose of preventative headache treatment \|  \|  \|  \|  \|  \| |
|  | 1. **Headache management I**   This section concerns your management of headache patients. |
| 38 | How important are the following results in your management of headache patients?   \|  \| Not important at all \| Not especially important \| Neutral \| Somewhat important \| Very important \| \| --- \| --- \| --- \| --- \| --- \| --- \| \| Prevention of headache episodes \|  \|  \|  \|  \|  \| \| Shorter duration of headache episodes \|  \|  \|  \|  \|  \| \| Pain relief during an episode \|  \|  \|  \|  \|  \| \| Improvement of headache-related coping strategies \|  \|  \|  \|  \|  \| \| An overall improvement of general health and well-being \|  \|  \|  \|  \|  \| |
| 39 | **Tension-type headache**: How often do you use the following treatment options in managing headache patients?   \|  \| Never \| Rarely \| Sometimes \| Often \| Always \| \| --- \| --- \| --- \| --- \| --- \| --- \| \| Manipulation \|  \|  \|  \|  \|  \| \| Mobilisation without an impulse \|  \|  \|  \|  \|  \| \| Activator \|  \|  \|  \|  \|  \| \| Drop treatment \|  \|  \|  \|  \|  \| \| Toggle recoil \|  \|  \|  \|  \|  \| \| Massage, myofascial techniques or trigger point treatment \|  \|  \|  \|  \|  \| \| Dry needling/ acupuncture \|  \|  \|  \|  \|  \| \| Electrotherapy (TENS, ultrasound, laser, etc.) \|  \|  \|  \|  \|  \| \| Jaw treatment \|  \|  \|  \|  \|  \| \| Exercises \|  \|  \|  \|  \|  \| \| Advice about stress management \|  \|  \|  \|  \|  \| \| Nutritional advice \|  \|  \|  \|  \|  \| \| Exercise advice \|  \|  \|  \|  \|  \| \| Advice about headache trigger factors \|  \|  \|  \|  \|  \| \| Other \|  \|  \|  \|  \|  \|   If you use ‘Other’, please state which other treatment options you use. |
| 40 | **Migraine**: How often do you use the following treatment options in managing headache patients?   \|  \| Never \| Rarely \| Sometimes \| Often \| Always \| \| --- \| --- \| --- \| --- \| --- \| --- \| \| Manipulation \|  \|  \|  \|  \|  \| \| Mobilisation without an impulse \|  \|  \|  \|  \|  \| \| Activator \|  \|  \|  \|  \|  \| \| Drop treatment \|  \|  \|  \|  \|  \| \| Toggle recoil \|  \|  \|  \|  \|  \| \| Massage, myofascial techniques or trigger point treatment \|  \|  \|  \|  \|  \| \| Dry needling/ acupuncture \|  \|  \|  \|  \|  \| \| Electrotherapy (TENS, ultrasound, laser, etc) \|  \|  \|  \|  \|  \| \| Jaw treatment \|  \|  \|  \|  \|  \| \| Exercises \|  \|  \|  \|  \|  \| \| Advice about stress management \|  \|  \|  \|  \|  \| \| Nutritional advice \|  \|  \|  \|  \|  \| \| Exercise advice \|  \|  \|  \|  \|  \| \| Advice about headache trigger factors \|  \|  \|  \|  \|  \| \| Other \|  \|  \|  \|  \|  \|   If you use ‘Other’, please state which other treatment options you use. |
| 41 | **Cervicogenic headache**: How often do you use the following treatment options with your headache patients?   \|  \| Never \| Rarely \| Sometimes \| Often \| Always \| \| --- \| --- \| --- \| --- \| --- \| --- \| \| Manipulation \|  \|  \|  \|  \|  \| \| Mobilisation without an impulse \|  \|  \|  \|  \|  \| \| Activator \|  \|  \|  \|  \|  \| \| Drop treatment \|  \|  \|  \|  \|  \| \| Toggle recoil \|  \|  \|  \|  \|  \| \| Massage, myofascial techniques or trigger point treatment \|  \|  \|  \|  \|  \| \| Dry needling/ acupuncture \|  \|  \|  \|  \|  \| \| Electrotherapy (TENS, ultrasound, laser, etc) \|  \|  \|  \|  \|  \| \| Jaw treatment \|  \|  \|  \|  \|  \| \| Exercises \|  \|  \|  \|  \|  \| \| Advice about stress management \|  \|  \|  \|  \|  \| \| Nutritional advice \|  \|  \|  \|  \|  \| \| Exercise advice \|  \|  \|  \|  \|  \| \| Advice about headache trigger factors \|  \|  \|  \|  \|  \| \| Other \|  \|  \|  \|  \|  \|   If you use ‘Other’, please state which other treatment options you use. |
|  | 1. **Headache management II**   This section concerns your treatment plans for headache patients |
| 42 | Indicate the average number of consultations you expect to see in a course of management of a new patient with the following headache types as their primary complaint   \|  \| 0-4 \| 5-7 \| 8-10 \| 11 or more \| \| --- \| --- \| --- \| --- \| --- \| \| Tension-type \|  \|  \|  \|  \| \| Migraine \|  \|  \|  \|  \| \| Cervicogenic headache \|  \|  \|  \|  \| |
| 43 | Indicate the average expected duration of a course of treatment for a new patient complaining of the following headache types   \|  \| 1-2 weeks \| 3-5 weeks \| 6-8 weeks \| 9 weeks or more \| \| --- \| --- \| --- \| --- \| --- \| \| Tension-type \|  \|  \|  \|  \| \| Migraine \|  \|  \|  \|  \| \| Cervicogenic headache \|  \|  \|  \|  \| |
| 44 | Indicate the average expected number of treatments per week, in a course of management of a new patient with the following headache types as their primary complaint.   \|  \| 1 time per week \| 2 times per week \| 3 times per week \| More than 3 times per week \| \| --- \| --- \| --- \| --- \| --- \| \| Tension-type \|  \|  \|  \|  \| \| Migraine \|  \|  \|  \|  \| \| Cervicogenic headache \|  \|  \|  \|  \| |
| 45 | In your experience, how effective is the treatment you provide as a chiropractor for the following types of headaches?   \|  \| Not effective \| Rarely effective \| Sometimes effective \| Often effective \| Difficult to discern \| \| --- \| --- \| --- \| --- \| --- \| --- \| \| Tension-type \|  \|  \|  \|  \|  \| \| Migraine \|  \|  \|  \|  \|  \| \| Cervicogenic headache \|  \|  \|  \|  \|  \| |
| 46 | Describe the most commonly experienced side-effects after treatment of patients with headaches |
| 47 | Do you have any comments to the answering of questions or your participation in this project? |
